# Evaluating the Efficacy of Coronary Sinus Reducer Implantation in the Management of Refractory Angina: A Systematic Review and Meta-Analysis

**DOI:** 10.7759/cureus.65662

**Published:** 2024-07-29

**Authors:** Gaurang H Suhagiya, Yoseph L Herpo, Darab Shuja, Aqsa A Butt, Muhammad Umar Mian, Sandipkumar S Chaudhari, Calvin R Wei, Adil Amin

**Affiliations:** 1 College of Medicine, Jiangsu University, Zhenjiang, CHN; 2 Internal Medicine, Hayat Medical College, Addis Ababa, ETH; 3 Internal Medicine, Services Hospital, Lahore, Lahore, PAK; 4 Internal Medicine, Allama Iqbal Medical College, Lahore, PAK; 5 Cardiothoracic Surgery, University of Alabama at Birmingham, Birmingham, USA; 6 Family Medicine, University of North Dakota School of Medicine and Health Sciences, Fargo, USA; 7 Research and Development, Shing Huei Group, Taipei, TWN; 8 Cardiology, Pakistan Navy Ship (PNS) Shifa, Karachi, PAK

**Keywords:** systematic review and meta-analysis, refractory angina, coronary sinus reducer implantation, efficacy, symptoms

## Abstract

The coronary sinus reducer (CSR), a minimally invasive device, has emerged as a promising alternative for improving myocardial perfusion in these patients. This meta-analysis evaluated the effectiveness of CSR implantation in patients with refractory angina. A comprehensive search of PubMed, EMBASE, and Web of Science databases identified 10 relevant studies with a pooled sample size of 799 patients. The analysis focused on changes in the Canadian Cardiovascular Society (CCS) classification score, Seattle Angina Questionnaire (SAQ) score, and six-minute walk distance (6MWD) from baseline to follow-up. Results showed significant improvements across all measured outcomes. CCS scores decreased significantly post-CSR implantation, indicating reduced angina severity. SAQ scores improved across all domains, including physical limitation, anginal stability, anginal frequency, treatment satisfaction, and quality of life, suggesting enhanced overall well-being. The 6MWD also increased significantly, reflecting improved functional capacity. These findings highlight CSR's potential as an effective treatment option for patients with refractory angina who have exhausted traditional therapies. CSR implantation appears to alleviate angina symptoms, improve quality of life, and enhance exercise tolerance. Future research should prioritize larger, multi-center randomized controlled trials to validate these findings. Long-term follow-up studies are needed to assess sustained benefits and potential risks.

## Introduction and background

Refractory angina is an important clinical condition, affecting up to 15% of individuals with severe ischemic cardiac illness [1]. It is a chronic, debilitating condition characterized by persistent chest pain due to coronary artery disease that remains uncontrolled despite optimal medical therapy and revascularization procedures [1]. This challenging form of angina significantly impacts patients' quality of life, limiting daily activities and increasing healthcare utilization [2]. It often occurs in patients with complex coronary anatomy, diffuse atherosclerosis, or those who have undergone multiple interventions [3]. The pathophysiology involves chronic myocardial ischemia, which standard treatments fail to address adequately. Management of refractory angina requires a multidisciplinary approach, focusing on symptom relief, improving functional capacity, and exploring novel therapies [4]. For this growing patient population, conventional anti-ischemic medications were the only available therapeutic option until recently. For individuals whose angina remains unresponsive to medication and adequate revascularization, new treatment alternatives have surfaced. These include implanting a coronary sinus reducer (CSR), spinal cord stimulation, and external counterpulsation [5]. 

For those with refractory angina, the CSR-a minimally invasive device-offers hope by improving blood flow to the heart muscle. This innovative device seeks to improve exercise capacity, relieve angina symptoms, and eventually improve the quality of life for patients who have exhausted traditional therapy choices by rerouting venous blood from the coronary sinus into the myocardium [6]. The European Society of Cardiology's most recent guidelines suggest CSR use in patients with refractory angina since it has been shown to be beneficial in reducing angina symptoms and enhancing quality of life (Class of Recommendation IIB) [7,8]. 

As research progresses, coronary sinus reducer implantation may become an increasingly important tool in the management of refractory angina, potentially reducing the need for repeated interventions and improving long-term outcomes for this challenging patient population. However, before this innovative intervention can be broadly adopted in clinical practice, its safety and efficacy must be rigorously evaluated. Accordingly, we present an extensive systematic review and meta-analysis, utilizing a substantial body of clinical evidence, to thoroughly assess the potential role of the coronary sinus reducer in managing refractory angina. Therefore, this meta-analysis aims to compare the effectiveness of CSR in improving symptoms in patients with refractory angina. 

## Review

Methodology 

The search strategy for this systematic review and meta-analysis was designed to comprehensively identify studies evaluating the effectiveness of coronary sinus reducer (CSR) implantation in patients with refractory angina. The search was conducted in the electronic databases PubMed, EMBASE, and Web of Science, covering the period from the inception of each database until June 26, 2024. A combination of keywords and Medical Subject Headings (MeSH) terms related to CSR and refractory angina were used. These search terms included “coronary sinus reducer”, “coronary sinus reduction”, “refractory angina”, and “angina”. The search was independently performed by two authors. Any disagreements regarding study inclusion were resolved through discussion and consensus, with a third author consulted if necessary to reach a final decision. The reference lists of all included studies were also reviewed to identify any additional relevant articles. This comprehensive approach ensured that the systematic review and meta-analysis included all pertinent studies assessing the impact of CSR on patients with refractory angina. 

Study Selection 

We included all studies that assessed the effectiveness of CSR in patients with angina by examining the improvement in the Canadian Cardiovascular Society (CCS) classification score and Seattle Angina Questionnaire (SAQ) score from baseline to follow-up in patients receiving CSR. The CCS is a grading system used to quantify the severity of angina pectoris, particularly in patients with coronary artery disease, with higher CCS classes indicating greater impairment and more frequent or severe angina symptoms [9]. The SAQ is a patient-reported tool that assesses the impact of angina on quality of life, including physical limitation, angina stability, angina frequency, treatment satisfaction, and disease perception. Higher scores on the SAQ reflect less severe symptoms and a more favorable impact on the patient's daily life [10]. We included original studies only. We excluded studies that did not include CSR. We also excluded studies that included non-adult patients and non-ischemic cardiac disease. We also excluded meta-analyses, reviews, case reports, and case series. 

All studies identified through our search strategy were imported into EndNote X9 for reference management. The initial screening of titles and abstracts was conducted by two independent authors to identify potentially relevant studies. This was followed by a thorough full-text review of the selected articles to determine their eligibility for inclusion in the systematic review and meta-analysis. Any disagreements between the two authors during the screening process were resolved through discussion and consensus, with a third author consulted if necessary to reach a final decision. 

*Data Extraction* 

Data extraction was meticulously performed by two independent authors to ensure accuracy and consistency. The following details were extracted from each included study: author name, year of publication, region of the study, sample size, follow-up duration, and baseline characteristics, such as age and gender of the participants. Key outcomes of interest, specifically the change in the Canadian Cardiovascular Society (CCS) classification score and Seattle Angina Questionnaire (SAQ) score from baseline to follow-up, were also extracted. Any discrepancies during the data extraction process were resolved through discussion and consensus, with a third author consulted if necessary to achieve a final agreement. 

Statistical Analysis 

We conducted a meta-analysis to evaluate changes in CCS and SAQ scores from baseline to follow-up. Effect sizes were pooled using a random-effects model to account for anticipated variations in study designs and populations. The results are reported as mean differences (MDs) with 95% confidence intervals (CI). A p-value of less than 0.05 was considered statistically significant. Heterogeneity between studies was assessed using the I-squared (I²) statistic, with values over 50% indicating significant heterogeneity. All meta-analyses were carried out using RevMan Version 5.4.1 (The Cochrane Collaboration, Oxford, United Kingdom). 

Results 

**Figure 1:**
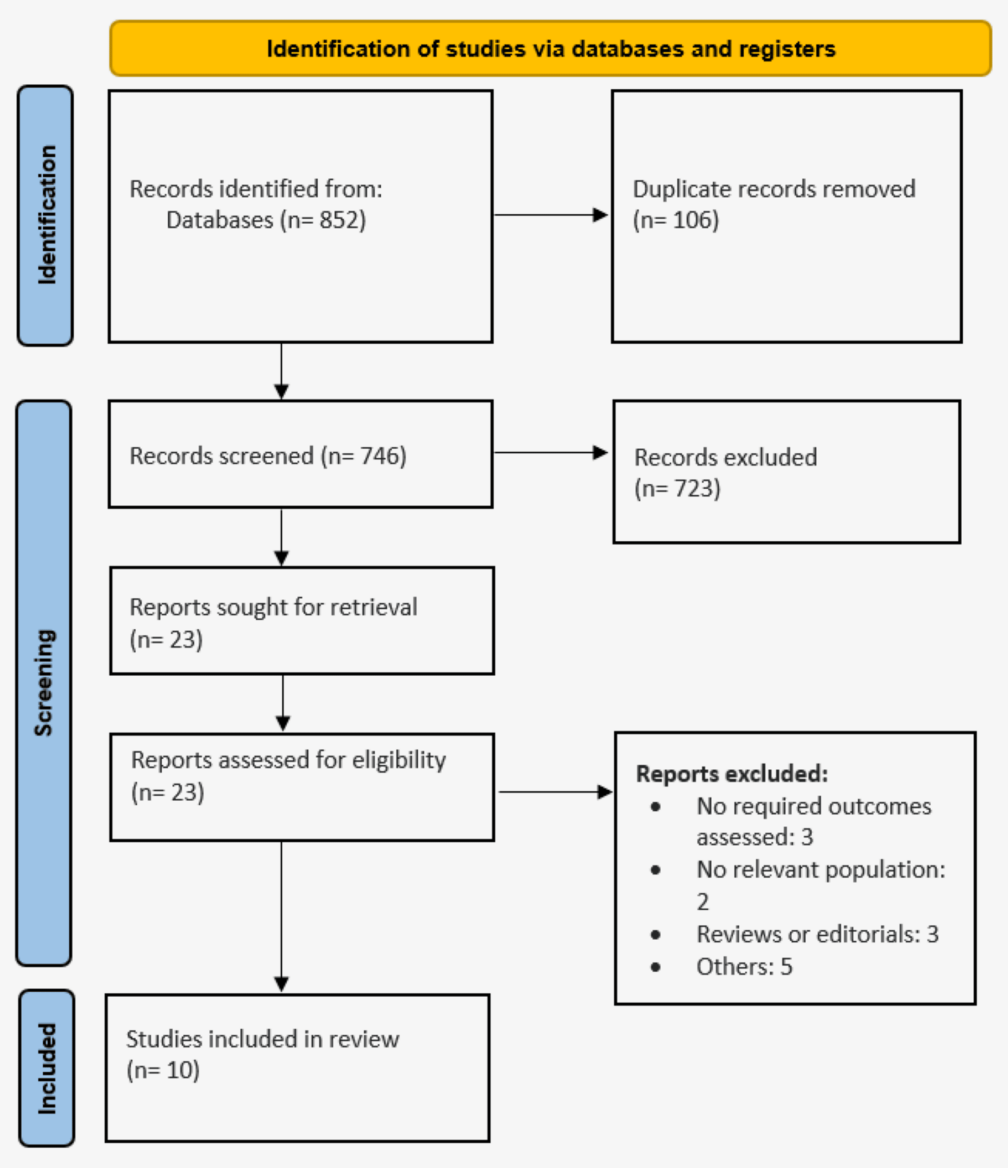
PRISMA flowchart showing the study selection process. PRISMA: Preferred Reporting Items for Systematic Reviews and Meta-Analyses.

Through the searching of databases, we identified 852 studies. Through the initial screening of 746 studies using titles and abstracts, we selected 23 studies for detailed screening based on pre-defined inclusion and exclusion criteria. Finally, 10 articles were included in this meta-analysis with a pooled sample size of 799 patients with refractory angina. Figure 1 shows the study selection process. Table 1 shows the characteristics of the included studies. Follow-up duration ranged from one month to 24 months. 

**Table 1:** Characteristics of included studies. NR: not reported.

Author	Year	Study design	Region	Sample size	Follow-up	Age	Males
D’Amico et al. [11]	2020	Prospective	Italy	187	24 Months	69.9	155
Foley et al. [12]	2024	Prospective	United Kingdom	25	6 Months	72	21
Konigstein et al. [13]	2018	Prospective	Israel	39	6 Months	66.8	40
Mrak et al. [14]	2021	Prospective	Slovenia	22	12 Months	71.5	21
Pontecelli et al. [15]	2019	Prospective	Italy	50	24 Months	NR	NR
Reis et al. [16]	2023	Prospective	Portugal	26	24 Months	71.8	20
Silvis et al. [17]	2021	Prospective	Netherlands	132	6 Months	66	100
Verheye et al. [18]	2021	Prospective	Multinational	180	24 Months	68.7	94
Vescovo et al. [19]	2022	Retrospective	Belgium	116	13 Months	69	88
Włodarczak et al. [20]	2023	Retrospective	Poland	22	1 Month	71.1	19

Effect of CSR on Change in CCS Score 

**Figure 2:**
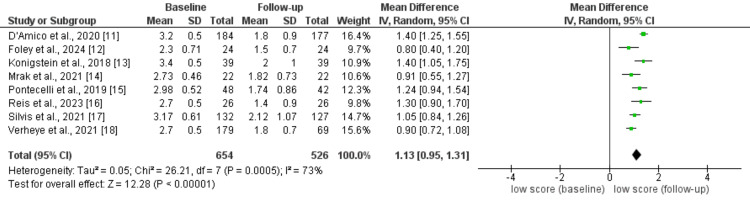
Effect of CSR on change in CSS. References [11-18]. CSR: coronary sinus reducer, CSS: Canadian Cardiovascular Society Classification Score.

A total of eight studies assessed the effect of CSR on change in CCS score from baseline. As shown in Figure 2, the CCS score was significantly lower after follow-up duration as compared to the baseline (MD: 1.13, 95% CI: 0.95 to 1.31), and this difference from statistically significant (p-value<0.001). It shows that CSR can play a vital role in reducing the severity of angina pectoris. Significant heterogeneity was reported among the study results (I-square: 73%). Significant heterogeneity is potentially due to the variable sample size and region where the study was conducted.

Effect of CSR on Change in SAQ 

The Seattle Angina Questionnaire (SAQ) includes five components: physical limitation, anginal stability, anginal frequency, treatment satisfaction, and quality of life. Table 2 demonstrates the effect of CSR on changes in the SAQ scores from baseline. The physical limitation score was significantly improved after follow-up compared to baseline (MD: -15.61, 95% CI: -19.89 to -11.34). Similarly, anginal stability was significantly better after follow-up (MD: -19.20, 95% CI: -27.37 to -11.03). Other aspects of the SAQ, including treatment satisfaction and quality of life, also showed significant improvements from baseline among patients receiving CSR. These results underscore the potential of CSR to enhance multiple dimensions of health-related quality of life in patients with refractory angina.

**Table 2:** Effect of CSR on SAQ outcomes. MD: mean difference; CI: confidence interval; CSR: coronary sinus reducer; SAQ: Seattle Angina Questionnaire.

Outcome	Number of studies	MD	95% CI	I-square
Physical limitation	7	-15.61	-19.89 to -11.34	59%
Anginal stability	6	-19.2	-27.37 to -11.03	86%
Anginal frequency	7	-25.09	-27.79 to -22.39	0%
Treatment satisfaction	6	-18.01	-28.83 to -7.19	40%
Quality of life	7	-28.99	-33.98 to -24.00	38%

Six-Meter Walking Distance (6MWD) 

**Figure 3:**
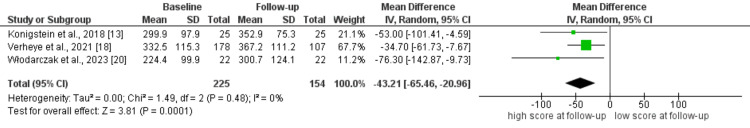
Effect of CSR on 6MWD. References [13,18,20]. CSR: coronary sinus reducer, 6MWD: six-minute walk distance.

A total of three studies assessed the effect of CSR on the change in six-minute walk distance (6MWD) score from baseline. As shown in Figure 3, the 6MWD was significantly greater after the follow-up duration compared to baseline in patients receiving CSR (MD: -43.21, 95% CI: -65.46 to -20.96), with this difference being statistically significant (p-value<0.001). These findings indicate that CSR can play a vital role in improving 6MWD in patients with refractory angina. Additionally, no significant heterogeneity was reported among the study results (I-square: 0%), suggesting consistency across the studies.

Discussion 

The findings highlight the significant impact of coronary sinus reducer (CSR) implantation on patients with refractory angina. CSR substantially reduced the severity of angina, improved multiple dimensions of health-related quality of life, and enhanced functional capacity as evidenced by significant improvements in the Canadian Cardiovascular Society (CCS) score, Seattle Angina Questionnaire (SAQ) scores, and six-minute walk distance (6MWD). These results underscore CSR's potential as a valuable therapeutic option. A similar meta-analysis performed by Theofilis et al. [21] reported that most individuals showed improvement in at least one class of CCS class after CSR, i.e., 75%, while 395 patients showed an improvement in CSS by at least two classes. 

The CSR has proven to be an effective new therapy for patients with refractory angina who were previously considered "no option" patients [22]. The COSIRA randomized, double-blind, sham-controlled clinical trial showed that, in addition to a significant placebo effect observed in both groups, narrowing of the CS provided greater angina relief compared to the sham procedure [18]. Another recently conducted randomized controlled trial (RCT) also reported that CSR did improve angina compared with placebo [12]. However, due to a lack of RCTs comparing CSR with placebo and other interventions and lacking. Therefore, in the future, more clinical trials are required to assess the efficacy of CSR to improve symptoms of angina. 

The 6MWT distance measures a patient's functional capacity and endurance, reflecting their ability to perform daily physical activities [23]. Improvement in 6MWT distance indicates enhanced exercise tolerance, cardiovascular fitness, and overall mobility [24]. The present meta-analysis showed that in patients with refractory angina, a greater 6MWT distance after treatment suggests that the intervention, such as CSR implantation, has effectively alleviated symptoms, thereby improving their physical performance and quality of life. 

Moreover, cardiopulmonary exercise testing (CPET) data in these patients showed an increased anaerobic threshold during follow-up after CSR implantation, while the peak respiratory exchange ratio remained unchanged [25]. These findings indicate that the enhanced exercise capacity observed in these patients was likely due to physiological changes induced by CSR implantation rather than merely improved motivation. According to our meta-analysis, patients who had CSR experienced a considerable improvement in their angina symptoms. This finding is especially significant in light of the subjective character of CCS class assessment, which has been linked in registries and other research to unfavorable outcomes like myocardial infarction and mortality [2]. By increasing myocardial perfusion, CSR considerably lessens the symptoms of angina by reducing ischemia and easing chest discomfort [12]. 

Notwithstanding these positive outcomes, it is critical to acknowledge the limitations of this meta-analysis. The character of the included studies is one of the main limitations. There was just one randomized controlled trial (RCT) that could be included, and its participant pool was somewhat limited. The remainder of the research was single-arm investigations. These elements, which include the RCT's small sample size and reliance on single-arm trials, have the potential to introduce bias and impair the findings' generalizability. Furthermore, one cannot completely rule out the placebo effect in single-arm research. As such, care should be taken while interpreting these results. 

Future studies should focus on conducting larger, multi-center randomized controlled trials (RCTs) to robustly validate the efficacy of coronary sinus reducer (CSR) in treating refractory angina. Long-term follow-up is crucial to assess sustained benefits and potential risks over extended periods. Comparative effectiveness research comparing CSR with other treatment modalities, such as enhanced external counterpulsation or spinal cord stimulation, would provide valuable insights into CSR's relative efficacy and safety profile. Furthermore, exploring patient-specific factors that may influence treatment outcomes, such as coronary anatomy and comorbidities, could optimize patient selection criteria and refine treatment protocols for CSR. 

## Conclusions

The coronary sinus reducer (CSR) shows promising results in managing refractory angina, as evidenced by significant improvements in CCS scores, SAQ scores, and six-minute walk distance. These findings suggest that CSR can effectively reduce angina severity, enhance quality of life, and improve functional capacity in patients with limited treatment options. However, the current evidence is limited by a lack of large-scale randomized controlled trials and potential placebo effects in single-arm studies. Future research should focus on conducting more robust clinical trials, exploring long-term outcomes, and comparing CSR with other treatment modalities. Despite these limitations, CSR appears to be a valuable therapeutic option for patients with refractory angina, warranting further investigation and consideration in clinical practice.
